# Aberrant Expression of Oncogenic and Tumor-Suppressive MicroRNAs in Cervical Cancer Is Required for Cancer Cell Growth

**DOI:** 10.1371/journal.pone.0002557

**Published:** 2008-07-02

**Authors:** Xiaohong Wang, Shuang Tang, Shu-Yun Le, Robert Lu, Janet S. Rader, Craig Meyers, Zhi-Ming Zheng

**Affiliations:** 1 HIV and AIDS Malignancy Branch, Center for Cancer Research, Nation Cancer Institute (NCI)/National Institutes of Health (NIH), Bethesda, Maryland, United States of America; 2 Nanobiology Program, Center for Cancer Research, Nation Cancer Institute (NCI)/National Institutes of Health (NIH), Bethesda, Maryland, United States of America; 3 Department of Obstetrics and Gynecology, Washington University School of Medicine, St. Louis, Missouri, United States of America; 4 Department of Microbiology and Immunology, Penn State University College of Medicine, Hershey, Pennsylvania, United States of America; University of Hong Kong, China

## Abstract

MicroRNAs (miRNAs) play important roles in cancer development. By cloning and sequencing of a HPV16^+^ CaSki cell small RNA library, we isolated 174 miRNAs (including the novel miR-193c) which could be grouped into 46 different miRNA species, with miR-21, miR-24, miR-27a, and miR-205 being most abundant. We chose for further study 10 miRNAs according to their cloning frequency and associated their levels in 10 cervical cancer- or cervical intraepithelial neoplasia-derived cell lines. No correlation was observed between their expression with the presence or absence of an integrated or episomal HPV genome. All cell lines examined contained no detectable miR-143 and miR-145. HPV-infected cell lines expressed a different set of miRNAs when grown in organotypic raft cultured as compared to monolayer cell culture, including expression of miR-143 and miR-145. This suggests a correlation between miRNA expression and tissue differentiation. Using miRNA array analyses for age-matched normal cervix and cervical cancer tissues, in combination with northern blot verification, we identified significantly deregulated miRNAs in cervical cancer tissues, with miR-126, miR-143, and miR-145 downregulation and miR-15b, miR-16, miR-146a, and miR-155 upregulation. Functional studies showed that both miR-143 and miR-145 are suppressive to cell growth. When introduced into cell lines, miR-146a was found to promote cell proliferation. Collectively, our data indicate that downregulation of miR-143 and miR-145 and upregulation of miR-146a play a role in cervical carcinogenesis.

## Introduction

Cervical cancer is one of the most common cancers in women worldwide, with an estimated global incidence of 470,000 new cases and approximately 233,000 deaths per year [1], [2]. Cervical cancer is the leading cause of death from cancer in many low-resource countries where widespread screening by cervical cytology is unavailable. The incidence is lower in developed countries as a consequence of cervical screening and of ongoing active health education programs. Cervical cancer rates in the United States were estimated as 10,370 new cases and 3,710 deaths in 2006 [3]. The causal relationship between high-risk HPV (HR-HPV) infection and cervical cancer has been well documented in epidemiological and functional studies. High-risk HPVs, such as HPV16, HPV18, and HPV31, have been detected in up to 99.7% of cervical squamous cell carcinomas and 94%–100% of cervical adeno- and adenosquamous carcinomas [4], [5]. The high-risk HPV oncoproteins, E6 and E7, contribute to cervical carcinogenesis by inactivating the cellular tumor suppressor proteins p53 and pRb, respectively [6]–[8].

miRNAs are regulatory, non-coding RNAs about 21–23 nucleotides in length and are expressed at specific stages of tissue development or cell differentiation, and have large-scale effects on the expression of a variety of genes at the post-transcriptional level. Through base-pairing with its targeted mRNAs, a miRNA induces RNA degradation or translational suppression of the targeted transcripts [9], [10]. Cellular miRNAs are transcribed by RNA polymerase II as long, capped, polyadenylated primary miRNA (pri-miRNA) transcripts bearing a stem-loop hairpin structure of ∼80-nts [11]. Mature miRNAs result from the processing of pri-miRNAs in two sequential cleavage steps mediated by two RNase III enzymes, Drosha and Dicer [12]. Drosha, in complex with DGCR8, cleaves the pri-miRNA at a specified distance (approximately 11 bp) from the stem–single stranded RNA junction in the nucleus to give a pre-miRNA of an ∼60-nt hairpin with a 2-nt 3′ overhang [13], [14]. After the pre-miRNA is exported to the cytoplasm by exportin 5 [15], [16], it is then recognized by Dicer, in complex with dsRBD-containing partner HIV-1 TAR RNA-binding protein (TRBP) [17], to remove the terminal loop, leaving a second 2-nt 3′ overhang [18]. The resulting 21- to 23-nt miRNA products contain a 2-nt 3′ overhang at each strand and act as the functional intermediates of RNAi that direct mRNA cleavage and translational attenuation. Although their biological functions remain largely unknown, recent studies suggest that miRNAs contribute to the development of various cancers [19] and might function as an important component of the cell's natural defense against viral infection [20]–[22]. It has been proposed that an unique miRNA expression profile for a particular cancer would be a useful biomarker for cancer diagnosis [23] and prognosis [24]. In this study, we used various approaches to profile miRNA expression from cervical cancer tissues and cervical cancer–derived cell lines as well as from HPV-infected vaginal keratinocytes. We demonstrate that a subset of miRNAs is significantly dysregulated in cervical cancers and pre-neoplastic lesions.

## Results

### miRNA expression profile in cervical cancer cell lines

**Figure 1:**
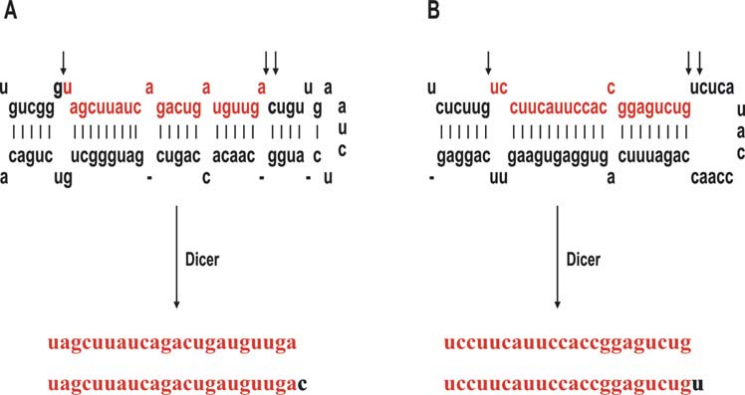
Wobble digestion of miRNAs by Dicer in CaSki cells. Arrows above the pre-miRNA indicate wobble-digestion sites on the pre–miR-21 in A and pre-miR-205 in B, producing products with additional nucleotides on the 3′ end. The sequences shown in red correspond to mature miRNA while that shown in black is the portion of pre-miRNA which is removed during processing.

**Figure 2:**
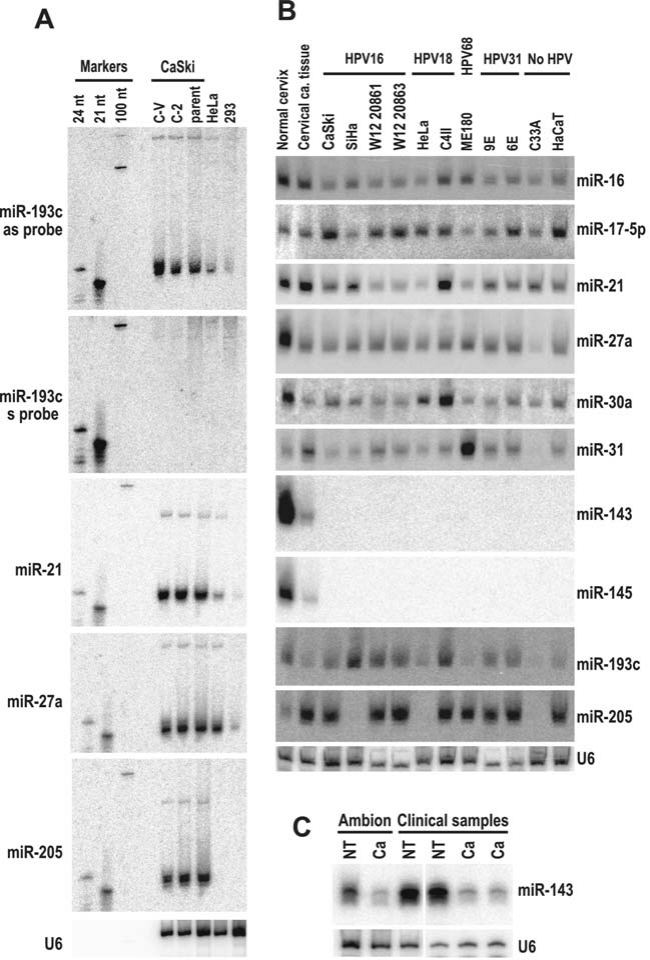
miRNA expression profile in cervical cancer cells determined by northern blot analysis. A. miR-193c expression in CaSki, HeLa, and 293 cells. CaSki and its subclones C-V and C-2 cells [72] were compared with HeLa (HPV18^+^) and 293 cells (HPV^−^) for expression of miR-193c by northern blotting. Total RNA (30 µg) from CaSki, HeLa, and 293 cells was separated in a 15% denaturing polyacrylamide gel, blotted onto a Gene Screen-Plus membrane, and then probed separately with a sense (s) or an antisense (as) miR-193c probe. Subsequently, the membrane was reprobed with antisense probes for miR-21, miR-27a, miR-205, or U6, respectively. B. miRNA expression in cervical cancer–derived cell lines. Total cell RNA (30 µg) was extracted from various cell lines with or without HPV infection. Northern blotting was used to examine the expression of 10 miRNAs from eight cancer cell lines, two W12 cell subclones, HaCaT cells, normal cervix, and cervical cancer tissues (Ambion). C. Downregulation of miR-143 in cervical cancer tissues. Total RNAs from two pairs of normal (NT) cervix vs cervical cancer (Ca) tissues from clinical samples and one pair of normal cervix and cervical cancer purchased from Ambion were compared for the expression of miR-143 by northern blotting. U6 was also blotted as an internal control for sample loading in panel A, B, and C.

To investigate the expression profiles of miRNAs in cervical cancer cells, we isolated total cell RNA from cervical cancer–derived CaSki C-2 cells, which contain ∼500 copies of the HPV16 genome per cell. The fractionated RNAs with sizes of 15–30 nts were cloned and sequenced based on a published protocol [25]. Overall, we cloned a total of 174 miRNAs from 363 cDNA clones which were categorized into 46 different miRNA species (Table 1), with miR-21, miR-24, miR-27a, and miR-205 being most abundant. No HPV16-derived miRNA was cloned in this screening, all the miRNAs were cellular. Interestingly, we found that some copies of the cloned miR-21 and miR-205 had heterogenous 3′ ends, with an extra C nucleotide on the 3′ end of 68% of the miR-21 copies and an extra U on the 3′ end of 33% of the miR-205 copies, presumably derived from wobble digestion of the pre-miRNAs by Dicer (Fig. 1). In addition, a new subspecies of miR-193, miR-193c (GenBank accession number: EF100863), was identified three times from the screening and was verified by northern blotting. The miR-193c subspecies differs from miR-193b by one nucleotide at position nt 21 and by the lack of three Us at the 3′ end (miR-193b, 5′-aacuggcccucaaagucccg**c**uuu-3′; miR-193c, 5′-aacuggcccucaaagucccg**a**-3′). The expression profile of the newly identified miR-193c which is 3-nt smaller than miR-193b was similar to that of miR-21 in HeLa cells and 293 cells, but less abundant than miR-27a. HeLa and 293 cells produced no detectable miR-205 as a doublet as seen in CaSki cells (Fig. 2A).

**Table 1:** miRNA expression profile in HPV16-positive CaSki C-2 cells by cDNA cloning.

miRNA names	Frequency of detection*	miRNA names	Frequency of detection*
miR-7	1	miR-92	1
miR-15a	2	miR-93	2
miR-16	7	miR-96	1
miR-17-5p	8	miR-106b	1
miR-19a	1	miR-130a	2
miR-19b	1	miR-130b	1
miR-20	3	miR-135b	1
miR-21	31	miR-141	3
miR-22	2	miR-151	1
miR-23a	5	miR-186	2
miR-24	10	miR-193b	1
miR-25	1	miR-193c	3
miR-26b	1	miR-203	2
miR-27a	29	miR-205	12
miR-27b	8	miR-301	2
miR-29a	3	miR-424	2
miR-29b	1	let-7a	6
miR-30a-3p	1	let-7b	2
miR-30a-5p	2	let-7d	2
miR-30d	1	let-7e	1
miR-31	3	let-7f	1
miR-33	1	let-7i	1
miR-34a	1		
miR-34c	1	**Total**	**174**

* Isolation frequency from 363 cDNA clones. CaSki C-2 cells stably express a hairpin-derived HPV16 E7 siRNA with no interfering function on HPV16 E7 expression (72). This screening also isolated 3 copies of the E7 siRNA.

Based on these cloning results, we further screened several available cervical cancer cell lines with or without HPV infection by northern blotting for the expression of 8 miRNAs with relatively high cloning frequency and 2 miRNAs (miR-143 and miR-145) which were not isolated in our cloning (Table 1). Eight cervical cancer cell lines; two isolates of the W12 cell line (20861 with integrated HPV16 genomes and 20863 with episomal HPV16 genomes) originally isolated from a cervical intraepithelial neoplasia (CIN) biopsy tissues [26] and HaCaT cells (an immortalized HPV-negative skin keratinocyte line) [27] were examined for the expression of the 10 miRNAs. A paired total RNAs from cervical cancer and adjacent normal cervical tissues commercially obtained from Ambion were also compared. All cell lines and cervical cancer tissues showed a similar miRNA expression profile. This miRNA profile differed from normal cervical tissues with reduced expression of miR-27a, miR-143, and miR-145 (Fig. 2B). In contrast, the cervical cancer tissue and the cell lines, except SiHa (HPV16^+^), HeLa (HPV18^+^), and C33A cells (HPV^−^), had increased expression of miR-205 when compared with normal cervical tissue (Fig. 2B). SiHa, HeLa, and C33A cells have no expression of miR-205. In all of the cell lines, expression of the miR-143 and -145 was less than what was observed in the cervical cancer tissue. Despite the presence of some differences in the expression of individual miRNA from one cell line to another, we were unable to specify an expression profile of those detected miRNAs in correlation to the presence of integrated or episomal HPV genomes. The downregulation of miR-143, which is derived from the same miRNA precursor of miR-145 [28], was further confirmed to be cancer-specific by comparing its expression in cervical cancer tissues to normal cervix (Fig. 2C).

### miRNA expression profile in normal and cancerous cervical tissues

**Figure 3:**
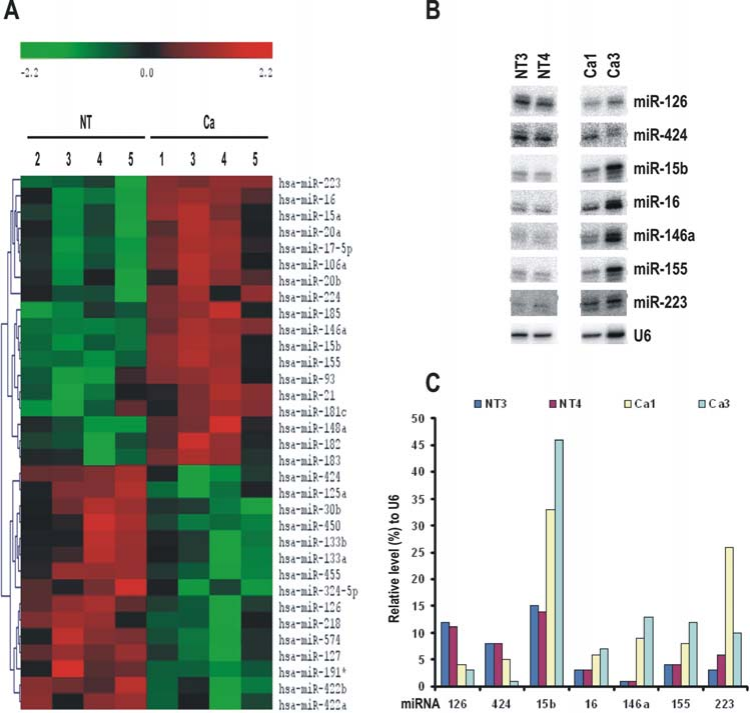
miRNA signatures in cervical cancer and normal cervical tissues by miRNA array analysis. Total RNA (5 µg) isolated from age-matched normal cervical tissues (NT) and cervical cancer tissues (Ca) were analyzed by miRNA array analysis. The cancer tissue 1, 3, and 5 were infected with HPV16 and the cancer tissue 4 was infected with HPV45. A. A clustering graph was created using a hierarchical method from those miRNAs with signal density of more than 1000 and shows increased (red) or decreased (green) expression (P<0.05) of individual miRNA from 4 age-matched normal and cancerous cervical tissues (Table S1). The column heading indicates individual sample code in the assay. Each row shows the expression pattern of individual miRNA as a log2-transformed expression ratio, with the most closely related expression joined by a branch and clustered by an average-linked algorithm. Branch lengths reflect degrees of similarity between miRNA expression patterns. Color scale on the top of the panel represents the degree of expression based on a calibrated ratio. Green indicates lower expression compared to the mean (zero), black indicates expression equal to the mean, and red indicates higher expression compared to the mean. B. Verification of miRNA expression profile by northern blotting. Total cell RNA (30 µg) isolated from each tissue was probed with individual antisense oligo to each indicated miRNA. An additional band in each blot indicates a wobble product from Dicer digestion. C. Bar graphs show the relative levels of the examined miRNAs normalized to U6 RNA in each tissue in panel B.

Since our cloning approach has a limitation in the isolation of low abundant miRNAs and northern blotting is limited by the probe chosen, the two methods may not be the best to distinguish a difference from one cell line to another in miRNA expression profile. To further investigate whether miRNAs are differentially expressed in cervical cancer tissues, we collected five pairs of age-matched normal and cancerous cervical tissues and compared their expression profiles using a miRNA array analysis containing 455 miRNAs. Only four sample pairs qualified for the final clustering analysis as determined by sample consistency analyses. An expression abundance analysis, based on a signal density ≥20,000, showed that both normal and cancerous cervical tissues had abundant expression of miR-23a, miR-23b, let-7a, let-7c, and let-7d, whereas high expression of miR-26a, miR-29a, miR-99a, miR-100, miR-125b, miR-143, miR-145, miR195, and miR-199a was only observed in normal cervical tissues, and high expression of miR-16, miR-21, miR-205, and let-7f was only observed in cervical cancer tissues, despite that relatively low levels of miR-16 and miR-21 were noticed from a homogeneous cell population in some cell lines (Fig. 2B). Expression of miR-143 and miR-145 showed more than 2.7-fold reduction in the cervical cancer tissues. Using a clustering analysis, we observed an significant increased expression of 18 miRNAs in cervical cancer and 15 miRNAs in normal cervix (Fig. 3A, miRNAs in red color) (P<0.05, t-test). For verification, a set of selected miRNA probes were used to perform a northern blot analysis for two normal and two cancer samples. We were able to confirm that the cancer tissues had reduced expression of miR-126 and miR-424, and increased expression of miR-15b, miR-16, miR-146a, miR-155, and miR-223 (Fig. 3B and 3C), after individual miRNA level in each sample was quantified and normalized to U6 expression.

### miRNA expression profile in HPV-infected, keratinocyte-derived raft tissues

**Figure 4:**
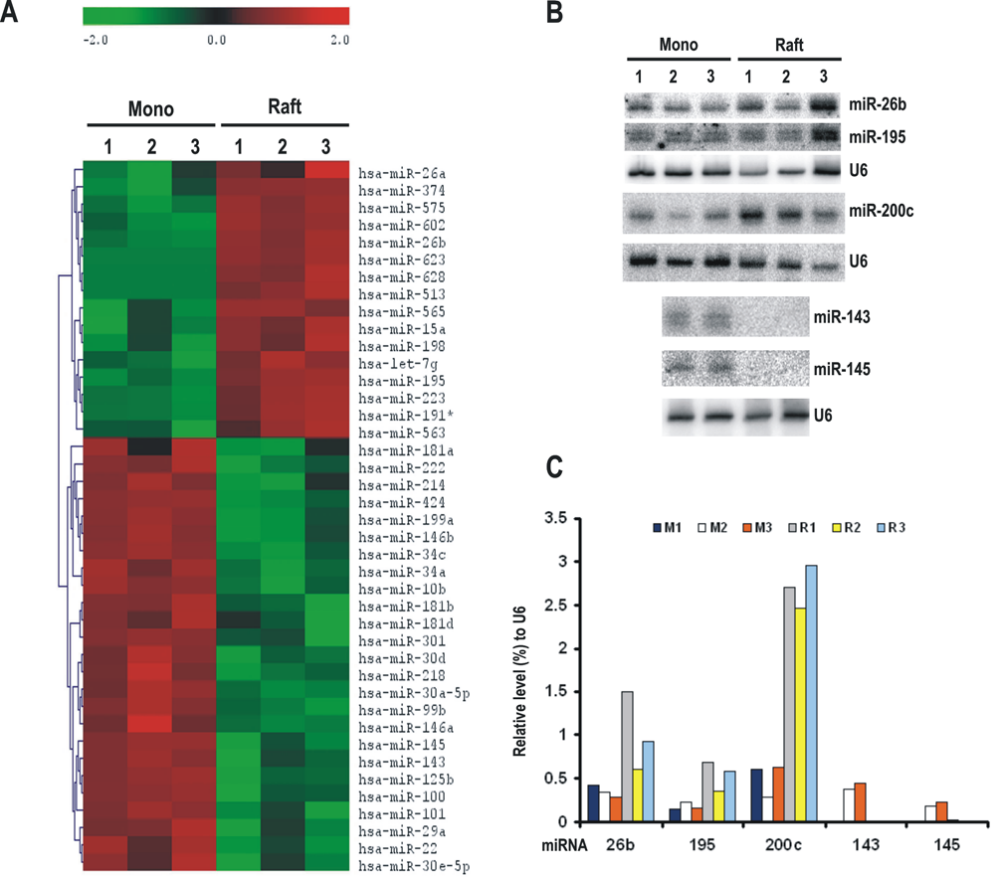
miRNA expression from undifferentiated keratinocytes in monolayers and stratified and differentiated keratinocytes in raft tissues. Raft tissues (cultures) with HPV18 infection were derived from human vaginal keratinocytes (HVKs) in monolayer cultures with HPV18 DNA transfection. Total cell RNA (5 µg each) extracted from each type of cells was compared in parallel for miRNA expression using miRNA array analyses. A. A clustering graph was created by a hierarchical method from those miRNAs with a signal density of more than 1000 obtained from three independent transfections in each group (Table S2). The graph shows decreased (green) or increased (red) expression (p<0.05) of individual miRNAs from undifferentiated HVKs in monolayers (Mono) versus stratified and differentiated HVKs in rafts. See other details in Fig. 3A. B. Verification of miRNA expression from undifferentiated HVKs in monolayers and stratified and differentiated HVKs in rafts by northern blotting. Total cell RNA (30 µg each) isolated from differentiated or undifferentiated HVKs was probed with selected miRNA probes. C. Bar graphs show the relative levels of the examined miRNAs normalized to U6 RNA in each sample in panel B. M = monolayer culture, R = raft.

**Figure 5:**
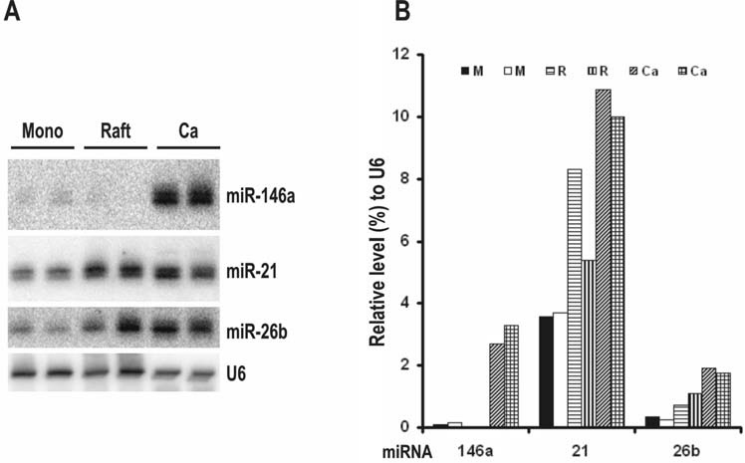
Upregulation of miR-146a is cervical cancer-specific. A. Total RNAs from HVKs in two rafts and two monolayer (Mono) cultures were compared with two cervical cancer (Ca) tissues for the expression of miR-146a by northern blotting. The expression levels of miR-21 and miR-26b were examined as miRNA controls. Small nuclear RNA U6 was also blotted as an internal control for sample loading. B. Bar graphs show the relative levels of the examined miRNAs normalized to U6 RNA in each sample in panel A. M = monolayer culture, R = raft.

To investigate whether the observed miRNA signatures in cervical cancer tissues are specific to cervical cancer, we examined miRNA expression in pre-neoplastic lesions. The lack of conventional cell cultures for HPV multiplication has largely restrained our understanding of the HPV life cycle and pathogenesis. However, infectious HPVs have been successfully produced from raft cultures derived from human keratinocytes [29], [30] and induction of pre-neoplastic lesions in raft tissues by HPV infection with the morphology similar to that of cervical intraepithelial neoplasia [31] makes this system of great advantage for our studies. Using miRNA array analyses, we compared the expression profiles of 455 human miRNAs in HPV18-infected primary human vaginal keratinocytes (HVKs) in monolayer cultures and in stratified and differentiated raft tissues. An expression abundance analysis, based on a signal density ≥20,000, showed that miR-21, miR-23a, miR-23b, miR-26a, miR-205, let-7c, and let-7f abundantly expressed in HVKs both in monolayer cultures and raft cultures. Three miRNAs abundantly expressed only in monolayer cultures (miR-24, miR-29a, and miR-221) and 11 miRNAs abundantly expressed only in the stratified and differentiated raft cultures (miR-27a, miR-27b, miR-200b, miR-200c, miR-203, miR-638, let-7a, let-7b, let-7d, let-7e, and let-7g). Using a clustering analysis, we further determined that 16 miRNAs were upregulated (Fig. 4A, raft in red) and 25 miRNAs were downregulated (Fig. 4A, raft in green) in the raft cultures when compared to the expression levels in the corresponding monolayer cultures (P<0.05, t-test). To confirm the observations, the paired samples were analyzed for miRNA expression by northern blotting using a set of specific miRNA probes (Fig. 4B and 4C). Quantitation of each miRNA level in each sample was normalized to U6 expression. We confirmed that the expression of miR-26b, miR-195, and miR-200c was increased in the raft cultures, but the expression of miR-143 and miR-145 was decreased (Fig. 4B and 4C). The increased abundance of miR-200c showing 67% more expression in rafts than in monolayers (P<0.08) by array analysis was verified by northern blotting. Although upregulation of miR-15a and miR-223 and downregulation of miR-218 and miR-424 were observed both in the rafts (pre-neoplastic lesions) and in cervical cancer tissues, the majority of the miRNA expression signatures showed no correlation between the two tissues (compare Fig. 4A to Fig. 3A). However, downregulation of miR-146a was noticed in the raft cultures rather than the upregulation seen in cervical cancer tissues and was further confirmed by northern blotting (Fig. 5), indicating that the upregulation of miR-146a expression is cervical cancer-specific. In contrast, a slight increased expression of miR-21 and miR-26b was observed in both the pre-neoplastic lesions (HPV-infected rafts) and cervical cancer tissues and thus their expression appears not cancer-specific (Fig. 5).

### Reduced miR-143 and miR-145 in cervical lesions and cervical cancer are suppressive in cervical cancer cell growth

**Figure 6:**
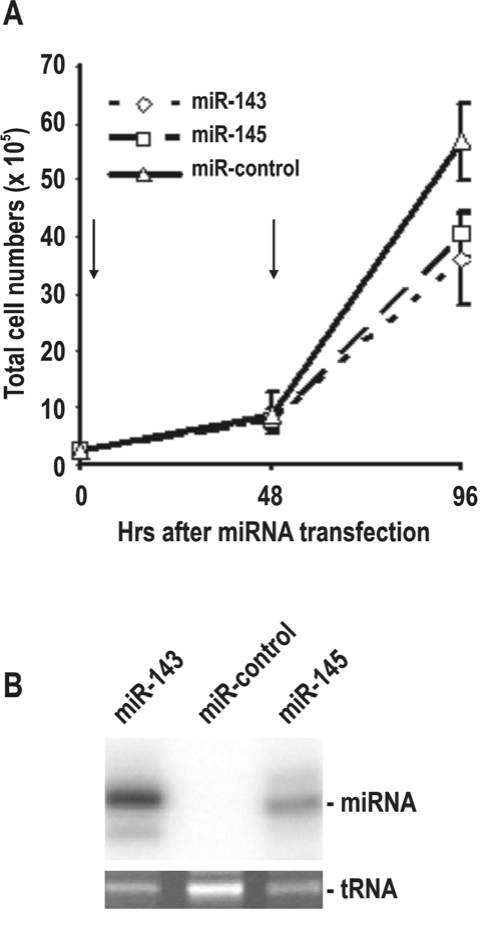
Overexpression of miR-143 and miR-145 suppresses HeLa cell growth. A. miR-143 and miR-145 are suppressive for HeLa cell growth. Arrows indicate the time points when the cells received a specific miRNA (30 nM) transfection. Viable cells in each group were counted at the indicated time points. Data represent the mean±SD of two experiments, each performed in triplicate. B. Relative levels of miR-143 and miR-145 in HeLa cells at 96 hrs after first transient transfection. Total cell RNA extracted was examined by miRNA ligation assay. A tRNA level in each sample was used as a loading control.

Having demonstrated a significant downregulation of miR-143 and miR-145 in cervical cancer and pre-neoplastic lesions, we investigated whether this downregulation is important in development of cervical cancer. To address this question, synthetic miR-143 and miR-145 precursors were transiently transfected into HeLa cells and the effect of overexpression of their mature miRNAs on HeLa cell growth was evaluated by cell counting. As shown in Fig. 6, both miR-143 and miR-145 were found suppressive (p<0.005 for miR-143 and p<0.008 of miR-145, *t*-test) to HeLa cell growth. This suppressive effect on the cell growth was not an immediate cell response; rather, two consecutive cell transfections with each miRNA at an interval of 48 hrs were needed. Data suggest that both miR-143 and miR-145 probably need to be downregulated in cervical cells for tumor progression.

### Increased miR-146a in cervical cancer promotes cell proliferation

**Figure 7:**
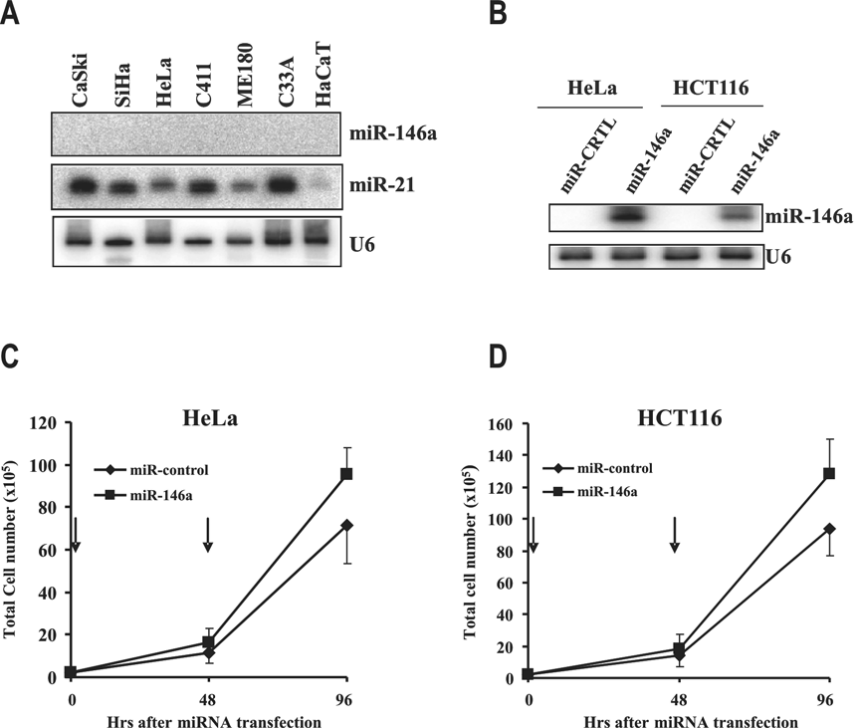
Overexpression of miR-146a promotes cell proliferation. A. No expression of miR-146a in cervical cancer-derived cell lines and in a human skin-derived keratinocyte line, HaCaT cells. Total cell RNA was extracted from individual cell line and examined for miR-146a and miR-21 expression by northern blotting. U6 in each sample served as sample loading control. B. Relative level of miR-146a in HeLa and HCT116 cells at 96 hrs after the first transfection. Total cell RNA extracted from each group was examined by northern blotting. C and D. miR-146a promotes cell proliferation. Arrows indicate the time points when the cells received transfection with miR-146a or miR-control (30 nM). Viable cells in each group were counted at the indicated time points. Data represent the mean±SD of two experiments, each performed in triplicate.

Considering a 6-fold increase of miR-146a expression in cervical cancer tissues, we hypothesized that a high level of miR-146a might play a role in cervical cancer cell growth. Our initial approach using anti-miR-146a inhibitors or using a morphonino-oligo to block miR-146a maturation to knock down miR-146a expression in cervical cancer cell lines failed to induce a phenotypic effect (data not shown). To our surprise, we found that all cervical cancer cell lines examined expressed no detectable miR-146a (Fig. 7A). Subsequently, miR-146a was introduced by transient transfection into HeLa cells, a HPV18^+^ cervical cancer cell line, and HCT116 cells, a colorectal cancer cell line (Fig. 7B). A slow, but significantly enhanced cell proliferation of both HeLa (p<0.009, *t*-test) and HCT116 (P<0.019, *t*-test) cells was noticed in the presence of exogenous miR-146a. By calculation of the cell counts from each group, we demonstrated that both HeLa cells and HCT116 cells containing miR-146a had a doubling time 2–3 hrs faster than that of the cells with miR-controls (Fig. 7C and 7D). This data suggests that miR-146a functions as a growth factor in cervical cancer.

## Discussion

In this study, we demonstrated miRNA expression profiles in cervical cancer and HPV infection-induced pre-neoplastic lesions in raft tissues. Although both cervical cancer tissues and HPV-infected raft tissues with pre-neoplastic lesions showed an upregulation of miR-15a and miR-223 and downregulation of miR-143, miR-145, miR-218, and miR-424, the majority of the miRNA expression showed no correlation between the two tissues and might delineate the disease progression. However, a high level of miR-146a expression was found to be cervical cancer-specific since its expression was down-regulated both in normal tissues and in HPV-infected raft tissues with pre-neoplastic lesions.

Finding of downregulation of miR-143 and miR-145 and upregulation of miR-146a in cervical cancer was in particular attractive for two reasons: (1) miR-143 and miR-145 are expressed from the same miRNA precursor [28] and are downregulated also in HPV-induced pre-neoplastic lesions, in our case in HPV-infected raft tissues, suggesting that their reduction take place in an early step before cancer development. Downregulation of miR-143 and miR-145 has been found in several other cancers, including colorectal cancer [32], B-cell lymphoma [33], and recently in cervical cancer [34], [35]. Thus, our finding is important for understanding the mechanisms by which miR-143 and miR-145 are involved in carcinogenesis. Inhibition by miR-143 and miR-145 of HeLa cell growth implies that miR-143 and miR-145 probably need to be downregulated in order for cancer growth. (2) Upregulation of miR-146a in cervical cancer tissues, but not in HPV-induced pre-neoplastic lesions or in cancer-derived cell lines indicates that miR-146 expression is cervical cancer-specific. Various studies show that miR-146a is a NF-kappaB-dependent gene [36]–[38]. Expression of miR-146a appears to vary in different cancer tissues in which an increased level of miR-146a was observed in Burkitts' lymphoma lines with EBV-LMP1 (latent membrane protein 1) expression [37], [38], but a decreased level in hormone-refractory prostate cancer [39] and papillary thyroid carcinoma [40]. In cervical cancer, upregulation of miR146a expression appears unrelated to any of HPV gene expression since its expression was even reduced in productively HPV-infected raft tissues. Interestingly, all cervical cancer cell lines and a skin keratinocyte line examined contains no detectable miR-146a, suggesting that miR-146a in cervical cancer tissues is upregulated by a factor missing in a homogenous cell population or is expressed by another type of cells in cancer tissues. Thus, we conclude that upregulation of miR-146a expression in cervical cancer is beneficial for cancer growth as introduction of miR-146a into cancer cells increased cell doubling time and promoted cell proliferation.

Approximately 800 human miRNAs have been predicted. Of these, more than 450 (http://microrna.sanger.ac.uk/sequences, 2006) have now been identified in the human [41], [42] [43], [44] , and their functions are beginning to be elucidated [45]. In this study, we cloned and identified 174 miRNAs which were grouped into 46 different miRNA species. A new subspecies of miR-193, miR-193c, was also cloned and identified from our miRNA library screening. Screening of 10 cell lines derived from cervical cancer or CIN tissues for expression of 10 miRNAs suggested that the presence or absence of integrated or episomal HPV genomes has no effect on the expression of analyzed miRNAs. Moreover, we were unable to identify a single HPV16-derived miRNA from HPV16^+^ CaSki cells by this approach, although other nuclear DNA viruses do encode miRNAs [46], including EBV [47]; KSHV [48]–[50]; HSV-1 [51], [52]; and SV40 [53]. A negative cloning for HPV16-derived miRNAs suggests that the integrated HPV16 genome in CaSki cells appears not to express viral miRNAs. Another study using differentiated HPV31^+^ LKP1 cells containing ∼50 copies of an episomal HPV31 genome per cell also failed to identify virus-derived miRNAs [54].

Recent reports have suggested that miRNAs contribute to a variety of cell functions [41] and are involved in the development of human cancers [19]. For example, miR-32 controls cell resistance to a retroviral infection [55]. A cluster of six miRNAs on human chromosome 13 is regulated by c-Myc, and the c-Myc–regulated miR-17-5p and miR-20a negatively modulate the expression of E2F1, a transcription factor [56]. miRNAs have been also characterized recently as potential oncogenes that promote the development of human B-cell lymphoma (miR-17-92 cluster) [57] and the proliferation and tumorigenesis of primary human cells (miR-372 and miR-373) by neutralizing p53-mediated CDK inhibition [58]. Another study suggested that overexpression of miR-17-5p, miR-20a, miR-21, miR-92, miR-106a, and miR-155 could be considered an miRNA signature of solid cancer [59]. In this report, 18 miRNAs were upregulated and 15 miRNAs were downregulated in cervical cancer tissues. The increased expression of miR-15b, miR-16, miR-146a, miR-155, and miR-223 that we observed in cervical cancer tissues has also been implicated in the development of other human cancers: miR-15 and miR-16 regulate apoptosis by targeting BCL2 [60] and their mutation has been associated with chronic lymphocytic leukemia [61]; miR-16 is also involved in control of cytokine RNA instability [62]; miR-146b levels are highly increased in papillary thyroid carcinoma [40]; miR-155 has recently been implicated in the development of lymphoblastic leukemia/high-grade lymphoma [63] and lung cancer [24] and in the regulation of human fibroblast angiotensin II type 1 receptor expression [64]; miR-223, along with the transcription factors C/EBPa and NFI-A, participates in regulation of granulocytic differentiation by suppressing the transcription of NFI-A mRNA [65].

Although mature miRNAs derived from their primary transcripts through Drosha and Dicer digestion in two separate trimming reactions contain a 2-nt 3′ overhang on each strand, Dicer digestion of the pre-miRNA is not always performed precisely. This was reflected in the miR-21 and miR-205 isolated in this study. We found that more than 68% of the miR-21 isolated from CaSki cells had an additional C at the 3′ end and 33% of the miR-205 isolated contained an extra U at the 3′ end, but no extra nucleotide was ever identified from the 5′ ends of either miRNA. When compared to the corresponding pre-miRNA sequence, the additional C on the miR-21 and the U on the miR-205 were found to be derived directly from the nucleotide 3′ to the cleavage site, suggesting that Dicer digestion has a wobble mechanism. This observation by small RNA cloning can be further verified as double bands for miR-21 (Fig. 5A and Fig. 7A) and for miR-205 (Fig. 2A and 2B) in northern blot analyses. Our findings are consistent with other reports obtained from biochemical analyses that Dicer uses a 3′ end counting rule and measures a distance of ∼22 nt from the 3′-terminus to cleave both strands of RNA, with 1- to 2-nt shifts in the preferred cleavage position [18], [66].

## Materials and Methods

### Cells, tissues, and raft cultures

Eight cervical cancer cell lines were used for the assays: HPV16^+^ CaSki and SiHa, HPV18^+^ HeLa and C4II, HPV68^+^ ME180 [67], and HPV31^+^ CIN612 6E and 9E cells. Two HPV16^+^ W12 subclones (20861 and 20863) originally isolated from a low-grade cervical lesion histologically diagnosed as CIN I [26] were also used for comparison. Among these cell lines, 20863 and 9E cells contain an episomal form of the HPV genome, and 20861 and 6E cells have an integrated HPV genome [68], [69]. A HPV-negative cervical cancer cell line, C33A [70], and a HPV-negative human keratinocyte line, HaCaT [27], were also included in this study. All cell lines, except for W12, 6E, and 9E cells, were grown in Dulbecco's modified Eagle's medium (DMEM) with 10% FBS at 37°C and 5% CO_2_. The W12 subclones and CIN612 6E and 9E were grown on pretreated NIH3T3 feeder cells [71]. Human primary vaginal keratinocytes and their derived raft cultures were prepared as described [30]. The stratified and differentiated keratinocytes in the raft culture epidermal layer were collected free from collagen (no fibroblasts) and frozen on dry ice for total cell RNA preparation. HCT116 cell line, a colorectal cancer cell line, was a gift from Dr. Bert Vogelstein of Johns Hopkins University and grown in McCoy's 5A medium with 10% FBS at 37°C and 5% CO_2_. Total cell RNA was prepared from each cell line using TRIzol Reagent (Invitrogen, Carlsbad, CA).

Age-matched normal cervix and cervical cancer tissues were obtained from anonymized excess cervical tissues that were no longer needed for diagnostic or clinical purposes. Use of these tissues was approved both by the Washington University Medical Center Human Studies Committee and by the NIH Office of Human Subjects Research. Each tissue was homogenized in an Eppedorf tube in 1 ml TRIzol Reagent using an electric homogenizer (Omni International, Marietta, GA) with a separate disposible probe for each tissue. The isolated RNA was dissolved in RNase-free water and stored in a −70°C freezer.

### miRNA cloning from cervical cancer cells

Cloning of miRNAs from cervical cancer cells was performed as described previously. Briefly, approximately 600 µg of total cell RNA prepared from HPV16-positive CaSki C-2 cells [a CaSki subclone cell line [72] was dissolved in RNA loading buffer II (Ambion, TX) and denatured at 75°C for 5 min. The RNA samples were then fractionated, along with ^32^P-labeled RNA oligos as size markers, in a 15% denaturing polyacrylamide gel containing 8 M urea. After the gel was exposed to an X-ray film, the small RNAs corresponding to the size range of the 15-nt and 30-nt oligos were cut from the gel, eluted with elution buffer (0.3 M NaCl, 0.5% SDS), and incubated overnight at 37°C. Subsequently, the small RNAs were ethanol precipitated and dephosphorylated with alkaline phosphatase and then ligated to a 5′-phosphorylated, ^32^P-labeled 3′ adapter (5′-rUrUrUAACCGCGAATTCCAG-L, L represents 3′ end protection with C7-amino modifiers, rU represents ribonucleotide) with T4 RNA ligase. After ligation, the ligated products were separated from unligated 3′ adapters in a 15% denaturing polyacrylamide gel and eluted overnight at 37°C from the gel slices with the elution buffer described above. After ethanol precipitation, the recovered small RNAs were phosphorylated with T4 polynucleotide kinase and ligated with a 5′ adapter (5′-ACGGAATTCCTCACTrArArA-3′, rA represents ribonucleotide) in the presence of T4 RNA ligase. The ligated RNAs were then separated from unligated 5′ adapters in a 15% denaturing polyacrylamide gel and eluted from the gel slices as described above. Reverse transcription was then conducted with a reverse primer (5′-GACTAGCTGGAATTCGCGGTTAAA-3′) and Superscript II (Invitrogen). The cDNA was amplified with the first PCR primer (5′-CAGCCAACGGAATTCCTCACTAAA-3′) and the reverse primer for 20 cycles of 45 sec at 94°C, 85 sec at 50°C, and 60 sec at 72°C. The PCR products were reamplified with a second pair of PCR primers (5′-GACTAGCTTGGTGCCGAATTCGCGGTTAAA-3′, 5′-GAGCCAACAGGCACCGAATTCCT CACTAAA-3′) for 10 cycles in the same conditions. The second PCR products were digested with *Ban*I, concatamerized with T4 DNA ligase, and cloned into a pCR2.1-TOPO vector. The bacterial colonies were first screened with PCR using two M13 primers (5′-GTAAAACGACGGCCAG-3′; 5′-CAGGAAACAGCTATGAC-3′). Colonies containing an insert from 400 bp to1000 bp in size were further grown and each plasmid DNA prepared was sequenced from both directions with M13 primers.

### µParaflo™ miRNA microarray assay

A microarray assay was performed using a service provider (LC Sciences, Houston, TX). The assay started with 5 µg of total RNA sample. The RNA was size-fractionated using a YM-100 Microcon centrifugal filter (GE Millipore, Billerica, MA), and the 3′ ends of the small RNAs (<300 nt) were extended with a poly(A) tail using poly(A) polymerase. An oligonucleotide tag was then ligated to the poly(A) tail for later fluorescent dye staining; two different tags were used for the two RNA samples in dual-sample experiments. Hybridization was performed overnight on a µParaflo microfluidic chip using a microcirculation pump [73], [74]. On the microfluidic chip, each detection probe consisted of a chemically modified nucleotide coding segment complementary to the target miRNA (455 human miRNA [hsa-miRNA] entries in version 8.1 from miRBase, http://microrna.sanger.ac.uk/sequences/) or other control RNAs and a spacer segment of polyethylene glycol to extend the coding segment away from the substrate. The detection probes were made by in situ synthesis using photogenerated reagent chemistry. The hybridization melting temperatures were balanced by chemical modifications of the detection probes. Hybridization was performed in 100 µL of 6× SSPE buffer (0.90 M NaCl, 60 mM Na_2_HPO_4_, 6 mM EDTA, pH 6.8) containing 25% formamide at 34°C. After hybridization, the miRNAs were detected by fluorescence labeling using tag-specific Cy3 and Cy5 dyes. Hybridization images were collected using a laser scanner (GenePix 4000B, Molecular Device) and digitized using Array-Pro image analysis software (Media Cybernetics). Data were analyzed by first subtracting the background and then normalizing the signals using a locally-weighted regression filter [75]. For two-color experiments, the ratio of the two sets of detected signals (log2 transformed, balanced) was calculated, and the significance of the differences were determined by *t*-test analysis; significantly different signals were those with p values less than 0.05. Multi-array normalization and clustering analysis were performed using a hierarchical method, with an average linkage and Euclidean distance metric. The clustering plots were generated using MultiExperiment Viewer software (v4.0, 2006) from The Institute for Genomic Research and were created from those miRNAs with a total signal density of more than 1000.

### Northern blot of small RNAs

Total cell RNA (30 µg) was resolved in a 12% or 15% (Fig. 2 only) denaturing polyacrylamide gel, transferred onto a GeneScreen-Plus Hybridization Transfer membrane (NEN PerkinElmer, Waltham, MA) in 0.5× TBE, and blotted with a ^32^P-labeled miRNA-specific oligo as described [72]. After blotting, the same membrane was stripped in 0.1× SSPE, 0.5% SDS at 85°C to 90°C for 20 min and prehybridized with hybridization buffer (Perfecthyb, Sigma) for 2 h and then hybridized with another probe overnight at 40°C. The membrane was washed in 2× SSPE containing 0.5% SDS, 0.5× SSPE containing 0.5% SDS, and 0.2× SSPE containing 0.1% SDS for 30 min each at 40°C, and then exposed to a PhosphorImager screen. The image was captured using a Molecular Dynamic PhosphorImager Storm 860 and analyzed with ImageQuant software. The oligo probes were designed based on individual miRNA sequence information deposited in miRBase (http://microrna.sanger.ac.uk). An antisense oligo of U6 snRNA (oST197, 5′-AAAATATGGAACGCTTCACGA-3′) was used to detect U6 snRNA from each sample as a loading control.

### miRNA ligation assay

miRNA ligation assay was performed using miRtect-IT™ miRNA Labeling and Detection Kit (USB, Cleveland, OH). Briefly, a detection oligo was 5′ end labeled with γ-^32^P-ATP and OptiKinase. The miRNA and the radiolabeled detection oligo were captured with a miRNA-specific bridge oligo and were then ligated with T4 DNA ligase. The ligated miRNA was separated on a 12% denaturing polyacrylamide gel and exposed to a PhosphorImager screen for 2.5 h. The image was captured and analyzed as described above.

### Transient miRNA transfection

Approximately 2.3×10^5^ HeLa and HCT116 cells were transfected with 30 nM of individual testing miRNA precursor or control-miRNA precursor (non-specific) purchased from Ambion. In each miRNA transfection, cell passage and miRNA transfection were conducted simultaneously using siPORT™ NeoFX™ Transfection Agent (Ambion) according to the manufacturer's instruction. After 48 h, the cells were counted, split, and transfected again with each corresponding miRNA with the same dose. After additional 48 h, the cells were counted and total RNA was extracted with TRIzol Reagent (Invitrogen). A student *t*-test was used for statistical analysis of cell counts.

## Supporting Information

Table S1 (0.08 MB XLS)

Table S2 (0.06 MB XLS)
